# Artificial Intelligence-Based Differential Diagnosis: Development and Validation of a Probabilistic Model to Address Lack of Large-Scale Clinical Datasets

**DOI:** 10.2196/17550

**Published:** 2020-04-28

**Authors:** Shahrukh Chishti, Karan Raj Jaggi, Anuj Saini, Gaurav Agarwal, Ashish Ranjan

**Affiliations:** 1 1mg Technologies Pvt Ltd Gurgaon India

**Keywords:** artificial intelligence, medical diagnosis, probabilistic modeling, Bayesian model, machine learning

## Abstract

**Background:**

Machine-learning or deep-learning algorithms for clinical diagnosis are inherently dependent on the availability of large-scale clinical datasets. Lack of such datasets and inherent problems such as overfitting often necessitate the development of innovative solutions. Probabilistic modeling closely mimics the rationale behind clinical diagnosis and represents a unique solution.

**Objective:**

The aim of this study was to develop and validate a probabilistic model for differential diagnosis in different medical domains.

**Methods:**

Numerical values of symptom-disease associations were utilized to mathematically represent medical domain knowledge. These values served as the core engine for the probabilistic model. For the given set of symptoms, the model was utilized to produce a ranked list of differential diagnoses, which was compared to the differential diagnosis constructed by a physician in a consult. Practicing medical specialists were integral in the development and validation of this model. Clinical vignettes (patient case studies) were utilized to compare the accuracy of doctors and the model against the assumed gold standard. The accuracy analysis was carried out over the following metrics: top 3 accuracy, precision, and recall.

**Results:**

The model demonstrated a statistically significant improvement (*P*=.002) in diagnostic accuracy (85%) as compared to the doctors’ performance (67%). This advantage was retained across all three categories of clinical vignettes: 100% vs 82% (*P*<.001) for highly specific disease presentation, 83% vs 65% for moderately specific disease presentation (*P*=.005), and 72% vs 49% (*P*<.001) for nonspecific disease presentation. The model performed slightly better than the doctors’ average in precision (62% vs 60%, *P*=.43) but there was no improvement with respect to recall (53% vs 56%, *P*=.27). However, neither difference was statistically significant.

**Conclusions:**

The present study demonstrates a drastic improvement over previously reported results that can be attributed to the development of a stable probabilistic framework utilizing symptom-disease associations to mathematically represent medical domain knowledge. The current iteration relies on static, manually curated values for calculating the degree of association. Shifting to real-world data–derived values represents the next step in model development.

## Introduction

The World Health Organization (WHO) advocates a minimum doctor:population ratio of 1:1000; although this prescribed ratio has been attained in most of the Western world, 44% of the WHO member states report less than 1 physician per 1000 patients [1]. The situation is particularly grim in South Asia and Africa, with a ratio as low as 0.01 physicians per 1000 individuals in Malawi [2]. The WHO estimates a global shortage of 12.9 million health care workers by 2035 [3]. Such acute shortages in the health care system necessitate the development of low-cost, deployable, and scalable tools that can be integrated into multiple health care delivery models.

Several machine-learning and deep-learning algorithms have been applied to facilitate clinical diagnosis, but such tools often require large clinical datasets for training. Lack of availability of such datasets and inherent problems such as overfitting often necessitate the development of innovative solutions. We here introduce a probabilistic model for medical diagnosis that has been developed from the ground up. This method utilizes a stable probabilistic framework that was subsequently adapted to local disease patterns in India. The model was developed and validated against differential diagnoses made by six doctors with respect to various symptom presentation scenarios.

## Methods

### Model Development

For model development, we focused on infectious diseases as a major contributor to patient morbidity and mortality in developing countries, with most patients presenting with fever as their primary symptom [4]. The 15 most common causes of fever in India (Multimedia Appendix 1) were identified through national epidemiological data and were independently verified by internal medicine and infectious disease specialists. These 15 diseases represent a bulk of the patient load and were used for developing the framework of the probabilistic model and subsequent accuracy testing. This approach allows for the construction of diagnostic tools that provide high levels of diagnostic accuracy while retaining the inherent scalability provided by mathematical constructs.

In a medical consultation, the objective of the doctor-patient interaction is to gather evidence to formulate a provisional diagnosis based on the presenting symptoms. The addition of every new symptom results in the probability of disease being modified; that is, the prior probabilities are updated with the addition of new evidence. Thus, the science behind reaching a clinical diagnosis mimics a probabilistic framework.

We developed a probabilistic model of diagnostic assessment, which was simplified to a multiclass classification problem. This required diseases to be distributed over a probabilistic distribution based on the presented evidence. The mathematical (Bayesian) interpretation of this distribution is a set of numbers corresponding to each possible disease. These numbers are representative of the probability of occurrence of a disease given the set of symptoms [5]. In this way, a list of differential diagnosis is generated.

To define the model, the following assumptions were considered: (1) medical history can be scientifically objectified, leading to definite universal symptom characteristics; (2) all possible diseases are included, which ensures a definite class of disease/condition; and (3) a single disease/condition is responsible for the set of presented symptoms. The mathematical framework for the model is described in detail in Multimedia Appendix 2.

### Validation

**Figure 1:**
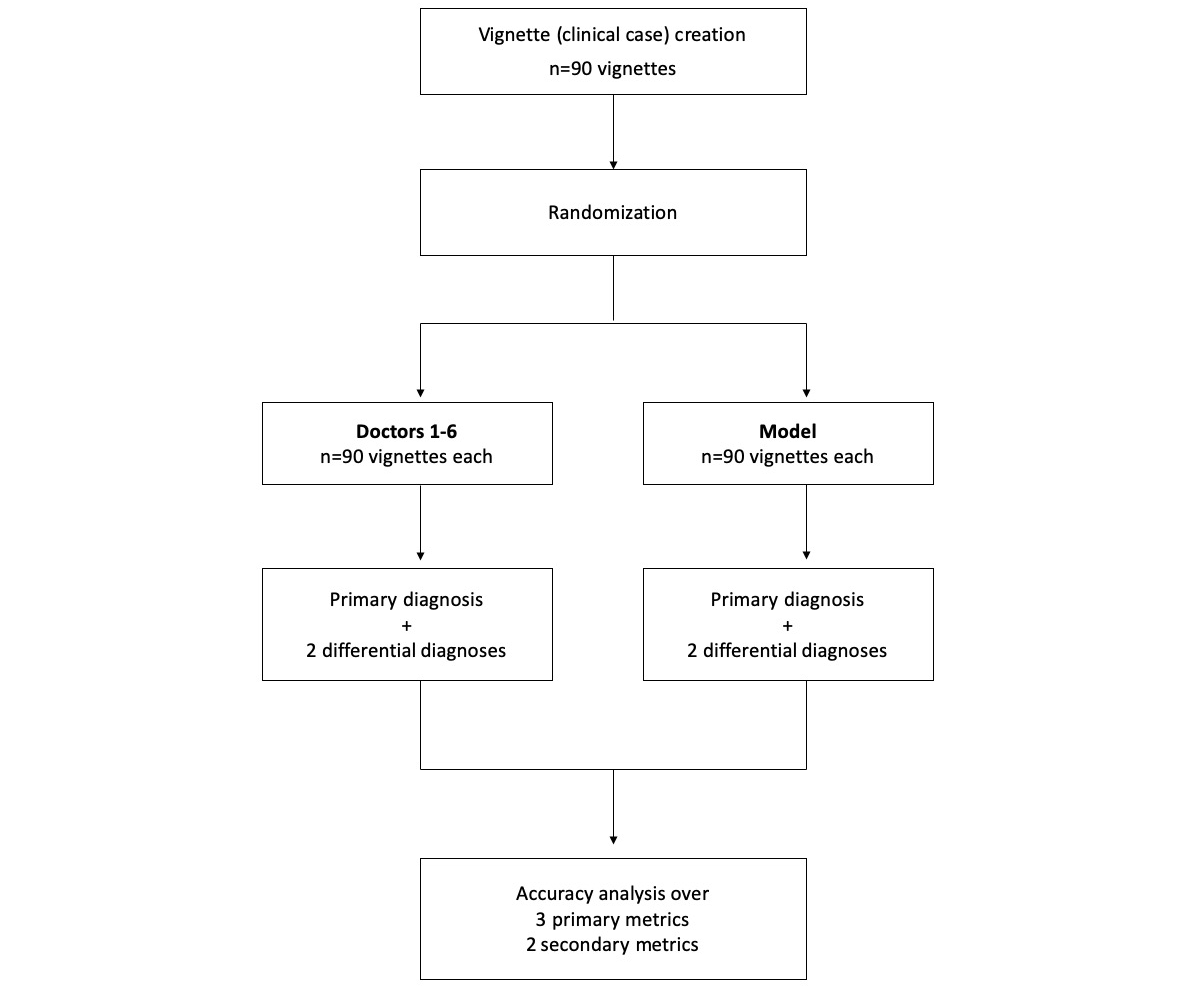
Study design.

An overview of the validation process is presented in Figure 1. Validation of the developed model was performed through a set of clinical vignettes (patient case summaries). To create the clinical vignettes, each of the 15 chosen diseases was stratified into three real-world clinical scenarios: highly specific disease presentation, moderately specific disease presentation, and nonspecific disease presentation. Two independent internal medicine specialists with a minimum clinical experience of 15 years each were entrusted with the creation of said vignettes, resulting in a total of 90 (15×3×2) clinical vignettes. The vignettes developed were subsequently verified by a third senior specialist to reduce any potential effects of selection bias.

Each clinical vignette was modeled on the standard medical history format containing patient demographic details (age, sex, and date of presentation), presenting complaints, present illness, and medical history, including family history and personal history. A primary diagnosis and two differential diagnoses with percentage surety values were provided in each vignette and were used for accuracy analysis. A sample clinical vignette is shown in Multimedia Appendix 3.

The clinical vignettes were randomized, assigned a code, and subsequently provided to 6 independent doctors of varying clinical experience (0-10 years). All participating doctors were provided with a brief detailing the clinical context prior to initiation of the study. For each vignette, the participating doctors were asked to provide one primary diagnosis and two differential diagnoses along with percentage surety values.

To calculate the performance of the probabilistic model, the clinical vignettes were fed into the model and were used to generate a ranked list of diagnoses with percentage surety values. Percentage surety values were utilized as an arbitrary representation of the level of confidence for each of the differential diagnoses, with the sum being 100% for each vignette. The top diagnosis from this list was assumed as the primary diagnosis for accuracy analysis.

### Accuracy Analysis

The performances of the model and the 6 doctors were compared against the assumed gold-standard diagnosis of the clinical vignettes under each of the three previously defined clinical scenarios: highly specific, moderately specific, and nonspecific disease presentation. Each of the chosen metrics provided a different perspective about the diagnostic accuracy.

The simplest method for accuracy analysis involves determination of the percentage of cases where the primary diagnosis (model or doctor) matches with the primary diagnosis of the clinical vignettes. Although this approach is useful in accuracy analysis for patients presenting with classical symptoms, it is not as suitable for patients with nonspecific presentations of the disease. Additionally, as all probabilistic models utilize only patient history for evaluation and do not take into account physical examination and relevant investigations, arriving at a single diagnosis is often medically unsound. Alternatively, top-3 accuracy, as an extension of the primary diagnostic accuracy, and standardized performance metrics such as precision and recall are useful in such scenarios, and were thus chosen as the main metrics for the current study.

The top-3 accuracy is an extension of the primary diagnostic accuracy and is defined as the percentage of cases where the list of three differential diagnoses developed contains the primary diagnosis of the clinical vignettes [6]. Thus, the top-3 accuracy denotes not only the diagnostic accuracy but also the safety of the model in situations faced by the patient. Precision, measured as the positive predictive value, is defined as the percentage of correct predictions (true positives/true positives+false positives). Precision is a measure of exactness and can be understood as the percentage of times a disease was correctly predicted among the total number of times its prediction was made. Precision was determined for each of the 15 individual diseases and an aggregate precision value was calculated by averaging over the frequency of each of the diseases. Recall, measured by sensitivity, is defined as the percentage of correct identification (true positives/true positives+false negatives). It is the percentage of times a disease was correctly predicted among the total number of times the disease was present. Recall was determined for each individual disease and an aggregate recall value was calculated. An upper-tailed *t* test was used for calculation of statistical significance.

## Results

For each of the defined metrics, an overall analysis of the performance of the model and doctors in comparison to the gold standard was performed. Additionally, comparative analysis was performed following stratification of the clinical vignettes based on the degree of specificity. Moving from highly specific to nonspecific vignettes denotes a progressive decrease in conclusive medical evidence available for diagnosis. In accordance, the performance of both the model and the participating doctors declined when moving down the spectrum of disease specificity.

**Figure 2:**
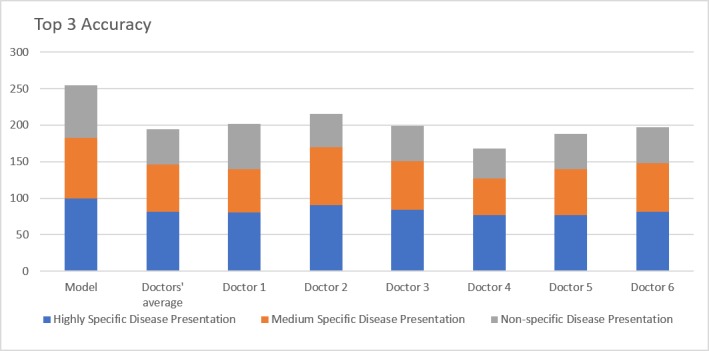
Top 3 accuracy. Comparison of model with doctors’ average and individual doctor's performances over three scenarios: highly specific, moderately specific and nonspecific disease presentations.

The top-3 accuracy of the model (Figure 2) was 85% in comparison to the doctor average of 67% (*P*=.002). The statistical significance of this advantage was retained across all three categories of clinical vignettes, 100% vs 82% (*P*<.001) for highly specific disease presentation, 83% vs 65% for moderately specific disease presentation (*P*=.005), and 72% vs 49% (*P*<.001) for nonspecific disease presentation.

**Figure 3:**
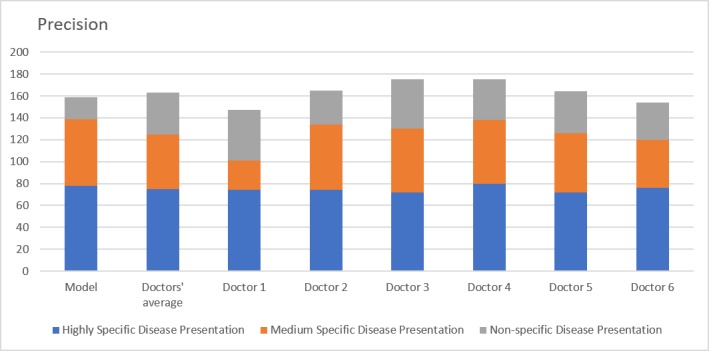
Precision. Comparison of model with doctors’ average and individual doctor's performances over three scenarios: highly specific, moderately specific and nonspecific disease presentations.

**Figure 4:**
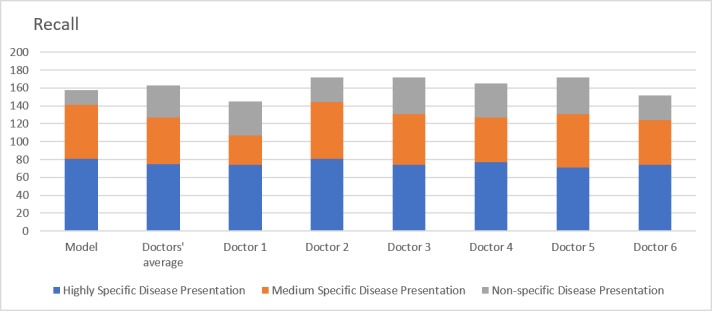
Recall. Comparison of model with doctors’ average and individual doctor's performances over three scenarios: highly specific, moderately specific and nonspecific disease presentations.

The precision for the model (Figure 3) was 62% in comparison to the doctor average of 60%. However, this difference was not statistically significant (*P*=.43). Stratification of the results revealed that the model performed better than doctors in highly specific vignettes (78% vs 75%, *P*=.04) and moderately specific vignettes (61% vs 50%, *P*=.09) but not for clinical vignettes with low specificity (20% vs 39%, *P*<.001). The overall recall for the model (Figure 4) was 53% in comparison to the doctor average of 56% (*P*=.27). Results obtained following segmentation of the clinical vignettes followed similar patterns, with the model faring better than doctors in highly specific (81% vs 75%, *P*=.008) and moderately specific (60% vs 52%, *P*=.13) vignettes, but not for nonspecific vignettes (17% vs 36%, *P*<.001).

**Figure 5:**
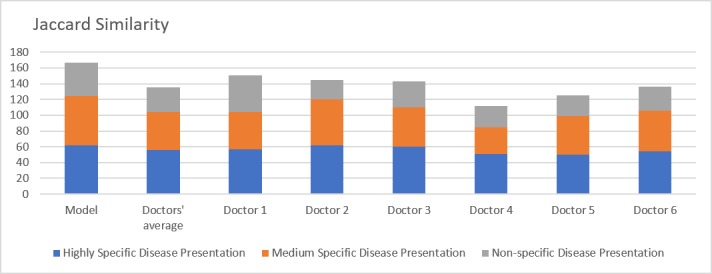
Jaccard Similarity. Comparison of model with doctors’ average and individual doctor's performances over three scenarios: highly specific, moderately specific and nonspecific disease presentations.

As discussed above, the top-3 accuracy, precision, and recall take only the primary diagnosis into account, and thus additional metrics such as the Jaccard similarity index and cosine similarity are of greater clinical relevance. Additional details about the chosen metrics are provided in Multimedia Appendix 4. Performance analysis on the basis of these metrics revealed that the model consistently outperformed doctors for both the complete set of clinical vignettes as well as in each of the three subcategories of vignettes. The overall performance of the model on the basis of the Jaccard similarity index was higher than that of the average doctor (56% vs 47%, *P*=.02). The performance differential between the model and the doctors was relatively narrow in the highly specific vignettes (62% vs 56%, *P*=.02) but widened considerably in the moderate (62% vs 48%, *P*=.008) and low (43% vs 31%, *P*=.01) specificity vignettes. An overview of the same is demonstrated in Figure 5.

**Figure 6:**
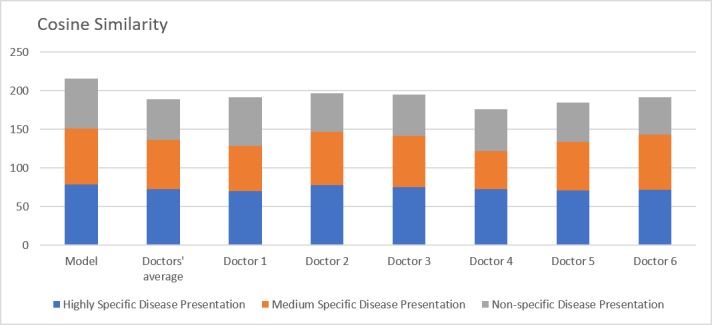
Cosine Similarity. Comparison of model with doctors’ average and individual doctor's performances over three scenarios: highly specific, moderately specific and nonspecific disease presentations.

The cosine similarity of the model (Figure 6) was 72% in comparison to the doctors’ average of 64% (*P*=.002). Performance following stratification of the vignettes revealed similar patterns to those found for the Jaccard similarity index, with a relatively narrow differential in the highly specific vignettes (79% vs 73%, *P*=.004) that widened in the moderate (72% vs 63%, *P*=.04) and low (65% vs 53%, *P*=.002) specificity of vignettes.

## Discussion

### Principal Findings

Historically, the art of medical diagnosis has relied upon the three pillars of patient history, physical examination, and investigative reports. Hampton et al [7] reported that a detailed and thorough patient history was sufficient to reach a clinical diagnosis in 83% of patients presenting to the medical outpatient department. This statistic is particularly relevant in settings where a patient’s physical examination and investigative tests cannot be performed. Advances in statistical analysis tools have allowed for the creation of probabilistic models serving as both diagnostic and prognostic tools for a myriad of clinical scenarios [8]. These models have been predominantly deployed in patients faced with the need for self-diagnosis, leading to the development of the common moniker “symptom checker” [9].

For the purpose of accuracy analysis, most authors advocate performance testing of both the model and doctors against an assumed gold standard. Evans et al [10] analyzed vignette methodology and concluded that vignettes are a powerful tool to study physicians’ clinical judgement, and can be strongly reflective of clinicians’ real-world behavior. This approach ensures that the accuracy analysis of the model is not unfairly hampered by errors in diagnosis by individual doctors. Additionally, as both the model and the doctors are fed the same information, over a statistically significant number, the quality of the clinical vignettes in question ceases to affect the accuracy analysis as the performance of both the model and doctors increases or decreases according to the level of information contained in the vignette. Razzouk et al [11] compared the performance of a decision support system for the diagnosis of schizophrenia disorders against an expert using 38 clinical vignettes prepared from outpatient charts. In a retrospective study, Ronicke et al [12] prepared clinical vignettes by extracting information from medical records, which were fed into the model (Ada DX) after anonymization. The second method of accuracy analysis involves a direct comparison of the model performance against diagnosis by the doctors, thereby assuming the doctors’ response as the gold standard. However, this approach does not take into account errors in judgement of the doctors themselves, which is currently estimated to vary between 10% and 15% [13]. This method also heavily relies on the assumption that the doctors participating in the study have sufficient clinical expertise, thus negating the effects of error over a statistically significant sample size. A recent study conducted by Berry et al [14] compared the diagnostic accuracy of the three most commonly used symptom checkers (WebMD, iTriage, FreeMD) in comparison to that of the doctors for patients presenting with abdominal pain, and found the accuracy of the top diagnosis to be 14.3%, with the top-3 accuracy being 36.7%. However, it is notable that only 49 patients (statistically insignificant) were included in the study and the level of experience of the practitioners was not taken into consideration [14].

Interestingly, once the sample size increases, the effects of diagnostic errors by individual doctors decreases, thereby presenting a fairer representation of the performance of the model. Bisson et al [15] compared the diagnostic accuracy of a web-based symptom checker for 328 patients (163 men and 165 women) presenting with knee pain, which revealed an accuracy of 58% for the model.

In the current study, the performance of both the model and doctors was compared to the assumed gold standard. In our opinion, this approach is statistically sound and should be considered for the primary performance evaluation of developed models, with a one-to-one analysis reserved for specific indications once the prerequisites of a statistically significant sample size and demonstrated clinical experience of the participating doctors have been met. The chosen metrics reflect not only the diagnostic accuracy but also the capability of the model to adapt to varying clinical scenarios and disease presentations [16].

The performance of the present model was superior to that of the panel of doctors across the entire gamut of chosen primary and secondary metrics. Several studies have failed to demonstrate such levels of clinical performance, with all trained models trailing in diagnostic accuracy achieved by clinicians. Semigran et al [9] used 45 clinical vignettes and reported 51.2% accuracy for the top-3 accuracy of an online symptom checker in comparison to 84.3% for doctors. A similar study conducted by Shen et al [6] for ophthalmologic diagnosis found the top-3 accuracy of the model to be a mere 38%. Davies et al [17] found that web-based symptom checkers listed degenerative cervical myelopathy as a differential diagnosis in only 45% of symptom composites tailored from 31 recognized symptoms in the literature. In addition, Ronicke et al [12] showed that their model (Ada DX) suggested a correct diagnosis in the top 5 suggestions in 53.8% of cases and as the top diagnosis in 37.6% of cases.

Although Razzouk et al [11] reported the model’s accuracy to be 66%-82% for diagnosing schizophrenia, it is important to mention that their model was constructed to diagnose only one disease and was evaluated for diagnosis of that particular disease.

### Strengths and Limitations

The current study represents the first probabilistic model that consistently outperformed trained medical professionals. This jump in diagnostic accuracy can by and large be attributed to building a model from the ground up. Various authors have utilized preconstructed or preconfigured models either directly or after small modifications. Shen et al [6] and Davies et al [17] used readily available web-based symptom checker tools such as WebMD, Healthtools, AARP, Healthline, or Netdoctor to study the accuracy of such models against the diagnostic performance of practicing physicians. However, in the present study, the probabilistic model was constructed from scratch. The mathematical framework was closely modeled to mimic the science behind arriving at a clinical diagnosis. Development of symptom-disease associations offers a novel approach to mathematically represent medical domain knowledge. Additionally, utilizing local epidemiological trends and disease profiles to develop these associations resulted in a high degree of accuracy. These symptom-disease relevance associations were manually curated by a team of doctors. This exercise was carried out over several iterations with extensive feedback from various medical experts. The model in the study represents the 6th iteration.

Delving deeper into the model performance after stratification of the clinical vignettes revealed some features that merit special mention. In cases where the clinical vignettes contain a large amount of specific clinical data (ie, highly specific and moderately specific disease presentation), the probabilistic model performed at par or better than the doctors across the gamut of chosen metrics (see Multimedia Appendix 5). However, this advantage was lost in the case of nonspecific disease presentation, with the model significantly trailing the panel of doctors in precision and recall values. These values serve as a reminder that even high-performing models fail in rare instances such as for patients with nonspecific disease presentations. Various studies have demonstrated similar results. In a review article, Mishra et al [18] lists unusual/atypical/silent disease presentation (nonspecific disease presentation) under the category “no fault errors.” They conclude that such errors are due to limitations of present medical knowledge and can only be reduced by furtherance of medical research and technological advancements. These findings are particularly relevant from a clinical context as they reinforce the fact that all such diagnostic tools should not be developed as a replacement for doctors but only to serve as clinical decision support systems. In a systematic review, Garg et al [19] reported that among 97 randomized and nonrandomized controlled trials studying the role of computerized clinical decision support systems in clinical practice, 64% of studies demonstrated improved practitioner performance. This approach will ensure standardization of care while retaining the inherent safety provided by a thorough clinical evaluation by a trained medical professional.

The present study suffers from a few limitations. First, the major limitation lies in the small sample size. Accuracy analysis was performed by comparing the performance of the model and the doctors over 90 clinical vignettes. Second, the present study was based on a small dataset (ie, patients presenting with fever in a developing country). This problem statement has been used to develop and subsequently test a probabilistic model. Further large-scale studies are required to prove the clinical relevance of this model across different clinical scenarios. The authors have attached the relevant study data in the appendices and encourage researchers to independently replicate the results on a statistically significant sample size. Lastly, a claim can be made that clinical vignettes might not represent a true visualization of the disease presentation, and accuracy analysis on actual patient data might prove to be beneficial [20].

### Future Scope

The current study represents a proof of concept of a probabilistic clinical decision support system. The present iteration of the model relies on static, manually curated values for calculating the degree of association. Shifting to real-world data–derived values represents the next step in model development. This migration would not only enhance diagnostic accuracy but also provide the ability to adapt to sudden changes in the disease environment in real time, an invaluable asset in disease epidemic prediction. Incorporation of investigative reports in the current framework—although challenging—is the key to a major jump in diagnostic accuracy. Accuracy testing for the current model has been performed through clinical vignettes that represent artificial textbook cases of disease presentations. Testing on real patient data would offer additional insights into the performance of the model.

### Conclusions

The present research demonstrates a drastic improvement over previously reported results that can be attributed to the development of the current model while keeping the local patient presentation and disease profile in mind instead of utilizing an off-the-shelf approach. This approach provides a greater degree of diagnostic accuracy than previous models. In addition, extensive involvement of practicing clinicians during the development phase is essential for the creation of a solution with demonstrable accuracy and clinical relevance. Importantly, these results are based on a relatively narrow dataset with the aim of developing a proof of concept. Therefore, additional large-scale clinical trials need to be conducted before these models can be deployed universally. In the interest of patient safety, the authors suggest positioning of all such tools as clinical decision support systems rather than as a substitute for trained medical doctors.

## Appendix

Multimedia Appendix 1 Fifteen most common causes of fever in India.

Multimedia Appendix 2 Mathematical framework.

Multimedia Appendix 3 Sample clinical vignette.

Multimedia Appendix 4 Secondary metrics.

Multimedia Appendix 5 Detailed results.

## Abbreviations

WHO: World Health Organization

## References

[1] (2019). Global Health Observatory (GHO) Data.

[2] WHO Global Health Workforce Statistics.

[3] (2013). Third Global Forum on Human Resources for Health Report.

[4] Herbrich R (2002). Learning Kernel Classifiers.

[5] Joyce J Stanford Encyclopedia of Philosophy.

[6] Shen C, Nguyen M, Gregor A, Isaza G, Beattie A (2019). Accuracy of a Popular Online Symptom Checker for Ophthalmic Diagnoses. JAMA Ophthalmol.

[7] Hampton JR, Harrison MJ, Mitchell JR, Prichard JS, Seymour C (1975). Relative contributions of history-taking, physical examination, and laboratory investigation to diagnosis and management of medical outpatients. Br Med J.

[8] Cahan A, Gilon D, Manor O, Paltiel O (2003). Probabilistic reasoning and clinical decision-making: do doctors overestimate diagnostic probabilities?. QJM.

[9] Semigran H, Linder J, Gidengil C, Mehrotra A (2015). Evaluation of symptom checkers for self diagnosis and triage: audit study. BMJ.

[10] Evans SC, Roberts MC, Keeley JW, Blossom JB, Amaro CM, Garcia AM, Stough CO, Canter KS, Robles R, Reed GM (2015). Vignette methodologies for studying clinicians' decision-making: Validity, utility, and application in ICD-11 field studies. Int J Clin Health Psychol.

[11] Razzouk D, Mari J, Shirakawa I, Wainer J, Sigulem D (2006). Decision support system for the diagnosis of schizophrenia disorders. Braz J Med Biol Res.

[12] Ronicke S, Hirsch MC, Türk E, Larionov K, Tientcheu D, Wagner AD (2019). Can a decision support system accelerate rare disease diagnosis? Evaluating the potential impact of Ada DX in a retrospective study. Orphanet J Rare Dis.

[13] Graber ML (2013). The incidence of diagnostic error in medicine. BMJ Qual Saf.

[14] Berry A, Cash B, Mulekar M, Wang B, Melvin A, Berry Bb (2017). Symptom Checkers vs. Doctors, the Ultimate Test: A Prospective Study of Patients Presenting with Abdominal Pain. Gastroenterology.

[15] Bisson LJ, Komm JT, Bernas GA, Fineberg MS, Marzo JM, Rauh MA, Smolinski RJ, Wind WM (2016). How Accurate Are Patients at Diagnosing the Cause of Their Knee Pain With the Help of a Web-based Symptom Checker?. Orthop J Sports Med.

[16] Brown JB (2018). Classifiers and their Metrics Quantified. Mol Inform.

[17] Davies BM, Munro CF, Kotter MR (2019). A Novel Insight Into the Challenges of Diagnosing Degenerative Cervical Myelopathy Using Web-Based Symptom Checkers. J Med Internet Res.

[18] Mishra D, Gupta P, Singh T (2017). Teaching for Reducing Diagnostic Errors. Indian Pediatr.

[19] Garg AX, Adhikari NKJ, McDonald H, Rosas-Arellano MP, Devereaux PJ, Beyene J, Sam J, Haynes RB (2005). Effects of computerized clinical decision support systems on practitioner performance and patient outcomes: a systematic review. JAMA.

[20] Randhawa H, Jiwa A, Oremus M (2015). Identifying the components of clinical vignettes describing Alzheimer's disease or other dementias: a scoping review. BMC Med Inform Decis Mak.

