# Supplementary material for: Artificial Intelligence-Based Differential Diagnosis: Development and Validation of a Probabilistic Model to Address Lack of Large-Scale Clinical Datasets
Source: J Med Internet Res. 2020 Apr 28;22(4):e17550. doi: 10.2196/17550 (PMC7218591; doi:10.2196/17550)
Supplement: Multimedia Appendix 1 [file jmir_v22i4e17550_app1.doc]

**Appendix 1: 15 most common causes of Fever in India**

| S.No | Disease |
| --- | --- |
| 1 | Bacterial Upper Respiratory Tract Infection |
| 2 | Viral Upper Respiratory Tract Infection |
| 3 | Sinusitis |
| 4 | Tonsillitis |
| 5 | Ear Infection |
| 6 | Pneumonia |
| 7 | Pulmonary Tuberculosis |
| 8 | Bronchitis |
| 9 | Gastro-enteritis |
| 10 | Hepatitis |
| 11 | Typhoid |
| 12 | Urinary Tract Infections |
| 13 | Dengue |
| 14 | Malaria |
| 15 | Chikungunya |
