# Supplementary material for: Artificial Intelligence-Based Differential Diagnosis: Development and Validation of a Probabilistic Model to Address Lack of Large-Scale Clinical Datasets
Source: J Med Internet Res. 2020 Apr 28;22(4):e17550. doi: 10.2196/17550 (PMC7218591; doi:10.2196/17550)
Supplement: Multimedia Appendix 2 [file jmir_v22i4e17550_app2.doc]

**Appendix 2: Mathematical Framework**

Symptoms (s) are defined as any medically relevant information extracted from the patient during a clinical evaluation. It includes patient demographic details, presenting complaints including characteristics such as duration of fever, character of pain etc. Values (v) are defined as the objectification of the response elicited from the patient, where the possibilities of a response are particular & restricted by the asked symptom v ∈ Values(s).

Evidence (E) is defined as a set of symptom-value pairs {sA:vA,sB:vB···} based on history of presenting illness. The universe of all possible symptom-value pairs {s:v} is E and every patient presents with a subset of E. In this way, the presented evidence E ⊂ E describe any consultation in a mathematical construct. This can be compared to history taking and subsequent data entry in the patients' health record.

Considering the set of all possible diseases C, out of which the patient is assumed to be suffering from a single one, with this framework, we can describe the probability of the disease outcome through relevance vectors.  Defining relevance weight vectors Rs:v ∈ R|C| for each s:v  ∈ E, that are parameters of the model M. And a function N acting on R|C|, that normalises the vector to unity, which allows comparison of disease diagnosis over different case scenarios.

E=∪i  si:vi

M(E) = P(c|E) = N(∑ii ∈ E Rsi:vi)

N(x) = softmax = exi / ∑i exi

Then the discriminating values in weights Rs:v incorporate the differential diagnosis.

This can be thought of as how relevant the appearance of a symptom is to determine various

diseases.  Intuitively, in a linear model summation of relevancies is the simplest way to objectify aggregation of evidence.

Differential Diagnosis

To compare the performance of model & medical personnel, their outcome should be brought to identical representation. So, we choose to represent differential diagnosis D of medics to a probability distribution Pmedic(c):

- Disease surety value from D is normalised and termed as probability
- Miscellaneous is treated as additional class
- Disease non-existent in D are assigned 0 in the distribution Pmedic(c)

Since, metrics & associated transform act functionally identical on Pmodel(c) and Pmedic(c), and, having transformed medics differential diagnosis D to probabilistic distribution Pmedic(c), subsequent metric evaluation refers both Pmodel(c), Pmedic(c) to P(c).

Miscellaneous Class

Miscellaneous is a special class defined to capture medics opinion of diagnosis, that is beyond the class of disease/condition we have defined. Firstly, it captures lack of surety in differential diagnosis, given the lack of physical examinations & other constraints. Secondly, it allows to entail disease/conditions that may be part of differential and absent in our scope of conditions C.
