# Supplementary material for: Artificial Intelligence-Based Differential Diagnosis: Development and Validation of a Probabilistic Model to Address Lack of Large-Scale Clinical Datasets
Source: J Med Internet Res. 2020 Apr 28;22(4):e17550. doi: 10.2196/17550 (PMC7218591; doi:10.2196/17550)
Supplement: Multimedia Appendix 3 [file jmir_v22i4e17550_app3.doc]

**Appendix 3: Sample Clinical Vignette**

**Vignette Code: 8A Vignette Number: 28**

**Assumed Diagnosis with surety value:** Pulmonary Tuberculosis 80% **Specificity: High**

**Differential Diagnosis with surety values:**

**1.** Chronic Bronchitis 15%

**2.** Miscellaneous 5%

**Vignette Code: 8A**

**Patient Details:**

1. Age: 40 years
2. Sex: Male
3. Date of presentation: 16^th^ July 2018

**Presenting Complaints (Brief):** Cough with expectoration since 3 months

Blood in sputum since 2 weeks

Fever since 1 week

**History of Present Illness (HOPI):** Cough with white to slight yellow sputum production since the last 3 months with a few episodes of blood in sputum since the last 2 weeks.

History of fever, on and off, since last 2 months; moderate to high grade associated with chills and rigors and associated evening rise of temperature.

History of loss of weight about 10 kg in the last 3 months.

**Family History:** History of tuberculosis in a family member.

**Past History:** Chronic smoker. No other significant past history.

**Personal History:** Low socio-economic status
