# Supplementary material for: Artificial Intelligence-Based Differential Diagnosis: Development and Validation of a Probabilistic Model to Address Lack of Large-Scale Clinical Datasets
Source: J Med Internet Res. 2020 Apr 28;22(4):e17550. doi: 10.2196/17550 (PMC7218591; doi:10.2196/17550)
Supplement: Multimedia Appendix 4 [file jmir_v22i4e17550_app4.doc]

**Appendix 4: Secondary Metrics**

a) Jaccard Similarity Index

The list of differential diagnosis i.e. primary diagnosis + 2 differential diagnoses was considered as a set and a set-to-set resemblance comparison was performed. The Jaccard coefficient, however, does not take into account the order of differential diagnosis or the percentage surety values attached to each differential. [S1] Thus differential diagnosis sets containing the correct diagnosis in an improper order cannot be identified on the basis of this metric. Similarly, it cannot differentiate between sets on the basis of surety values, a differentiation which is imperative in a clinical setting.

b) Cosine Similarity

Cosine Similarity was used to compare the similarity between the predicted differential diagnosis and the assumed gold standard (clinical vignette) taking both the ranking and the surety values into consideration.[S2] It was derived as the dot product of the normalised surety vectors of the predicted differential diagnosis & the labelled gold standard, thus representing the most clinically relevant metric.

Supplementary Material References:

S1) Tan PN, BoSteinbach M, Kumar V. Introduction to Data Mining. 2nd ed. Boston, MA: Addison-Wesley; 2018. (online) Available from: [http://www-users.cs.umn.edu/~kumar/dmbook/ [Accessed 22 Apr. 2019]](http://www-users.cs.umn.edu/~kumar/dmbook/).

S2) Singhal A. Modern Information Retrieval: A Brief Overview. In: Bulletin of the IEEE Computer Society Technical Committee on Data Engineering. 2001;24(4):35-43.
