# Supplementary material for: Artificial Intelligence-Based Differential Diagnosis: Development and Validation of a Probabilistic Model to Address Lack of Large-Scale Clinical Datasets
Source: J Med Internet Res. 2020 Apr 28;22(4):e17550. doi: 10.2196/17550 (PMC7218591; doi:10.2196/17550)
Supplement: Multimedia Appendix 5 [file jmir_v22i4e17550_app5.doc]

**Appendix 5: Detailed Results**

Overall Vignettes (n=90)

| **Metric** | **Model** | **Doctor 1** | **Doctor 2** | **Doctor 3** | **Doctor 4** | **Doctor 5** | **Doctor 6** |
| --- | --- | --- | --- | --- | --- | --- | --- |
| Top 3 Accuracy | 85 | 68 | 80 | 67 | 57 | 63 | 64 |
| Precision | 62 | 47 | 60 | 64 | 64 | 65 | 58 |
| Recall | 53 | 48 | 63 | 58 | 56 | 58 | 51 |
| Jaccard Similarity | 56 | 49 | 58 | 48 | 37 | 42 | 45 |
| Cosine Similarity | 72 | 64 | 69 | 66 | 59 | 62 | 64 |

Highly Specific Disease Presentation (n=30)

| **Metric** | **Model** | **Doctor 1** | **Doctor 2** | **Doctor 3** | **Doctor 4** | **Doctor 5** | **Doctor 6** |
| --- | --- | --- | --- | --- | --- | --- | --- |
| Top 3 Accuracy | 100 | 80 | 90 | 84 | 77 | 77 | 81 |
| Precision | 78 | 74 | 74 | 72 | 80 | 72 | 76 |
| Recall | 81 | 74 | 81 | 74 | 77 | 71 | 74 |
| Jaccard Similarity | 62 | 57 | 62 | 60 | 51 | 50 | 54 |
| Cosine Similarity | 79 | 70 | 78 | 75 | 73 | 71 | 72 |

Medium Specific Disease Presentation (n=30)

| **Metric** | **Model** | **Doctor 1** | **Doctor 2** | **Doctor 3** | **Doctor 4** | **Doctor 5** | **Doctor 6** |
| --- | --- | --- | --- | --- | --- | --- | --- |
| Top 3 Accuracy | 83 | 60 | 80 | 67 | 50 | 63 | 67 |
| Precision | 61 | 27 | 60 | 58 | 58 | 54 | 44 |
| Recall | 60 | 33 | 63 | 57 | 50 | 60 | 50 |
| Jaccard Similarity | 62 | 47 | 58 | 50 | 34 | 49 | 52 |
| Cosine Similarity | 72 | 59 | 69 | 67 | 49 | 63 | 71 |

Non-specific Disease Presentation (n=30)

| **Metric** | **Model** | **Doctor 1** | **Doctor 2** | **Doctor 3** | **Doctor 4** | **Doctor 5** | **Doctor 6** |
| --- | --- | --- | --- | --- | --- | --- | --- |
| Top 3 Accuracy | 72 | 62 | 45 | 48 | 41 | 48 | 49 |
| Precision | 20 | 46 | 31 | 45 | 37 | 38 | 34 |
| Recall | 17 | 38 | 28 | 41 | 38 | 41 | 28 |
| Jaccard Similarity | 43 | 47 | 25 | 33 | 27 | 26 | 30 |
| Cosine Similarity | 65 | 63 | 50 | 53 | 54 | 51 | 49 |
